# Crystal structure of a μ-oxo vanadium(V) dimer coordinated by a salan ligand

**DOI:** 10.1107/S2056989025008631

**Published:** 2025-10-14

**Authors:** Isha A. Kallingal, Anais A. Alvarado, S. Chantal E. Stieber, Alex John

**Affiliations:** a Department of Chemistry & Biochemistry Cal Poly Pomona, 3801 W Temple Ave Pomona CA 91768 USA; University of Neuchâtel, Switzerland

**Keywords:** crystal structure, vanadium(V), *μ*-oxo dimer, salan ligand, octa­hedral geometry

## Abstract

A *μ*-oxo vanadium(V) dimeric complex, μ-oxido-bis­[(2,2′-{[ethane-1,2-diylbis(aza­nedi­yl)]bis­(methyl­ene)}diphenolato)oxidovanadium(V)],[V_2_(C_16_H_18_N_2_O_2_)_2_O_3_] (**1**), was crystallized by slow evaporation from an ethanol solution. The *μ*-oxo dimer crystallizes in the monoclinic space group *C*2/*c* where the salan ligand **1a** coordinates to the vanadium center in a κ^2^*N*,κ^2^*O* fashion, forming a distorted octa­hedral geometry. The bridging oxo ligand lies on a crystallographic twofold axis. The unit cell consists of four mol­ecules of **1** that are linked by C—H⋯·π_arene_ inter­actions as well as intra­molecular hydrogen bonding.

## Abstract 2

A *μ*-oxo vanadium(V) dimeric complex, μ-oxido-bis­[(2,2′-{[ethane-1,2-diylbis(aza­nedi­yl)]bis­(methyl­ene)}diphenolato)oxidovanadium(V)], was synthesized and structurally characterized.

## 1. Chemical context

Vanadium compounds have been a subject of sustained investigation due to the varied oxidation states displayed by vanadium, which render unique reactivity to its compounds (Hu *et al.*, 2023 ▸). In nature, vanadium is found in enzymes such as nitro­genases, nitrate reductases, and haloperoxidase *etc* (Hu *et al.*, 2012 ▸; Eady, 1996 ▸; Butler & Walker, 1993 ▸). Complexes featuring a vanadium IV or V center with an oxovanadium (VO^2+^, VO^3+^) or dioxovanadium (VO_2_^+^) core along with their *μ*-oxo or *μ*-alkoxo-bridged dimers are well-known and are commonly encountered in biochemical, pharmacological, and catalytic studies. Synthetic applications of such complexes include epoxidations, alcohol oxidations, sulfoxidation, hydrogenation, cross-coupling reactions, oligomerization and polymerization, de­oxy­dehydration etc (Drzeżdżon *et al.*, 2024 ▸; Patra *et al.*, 2021 ▸; Hasnaoui *et al.*, 2020 ▸; Hossain *et al.*, 2019 ▸; Gopaladasu & Nicholas, 2016 ▸; Chapman & Nicholas, 2013 ▸; Hosseini Monfared *et al.*, 2011 ▸; da Silva *et al.*, 2011 ▸; Hoppe & Limberg, 2007 ▸; Maity *et al.*, 2007 ▸; Baran, 2000 ▸). Modular ligands have been demonstrated to tune the structure and reactivity of the metal center in these oxovanadium and dioxovanadium complexes (Gopaladasu & Nicholas, 2016 ▸; Adão *et al.*, 2009 ▸). Our group is currently exploring the unique reactivity of (di)oxovanadium complexes stabilized by salan ligands in contemporary oxidation and reduction reactions. Ligated metal complexes are readily accessible and offer a platform to establish structure–activity relationships to gain deeper understanding into catalytic processes (Wagner *et al.*, 2024 ▸; Dereli *et al.*, 2018 ▸; Steelman *et al.*, 2014 ▸, 2013 ▸).

## 2. Structural commentary

**Figure 1:**
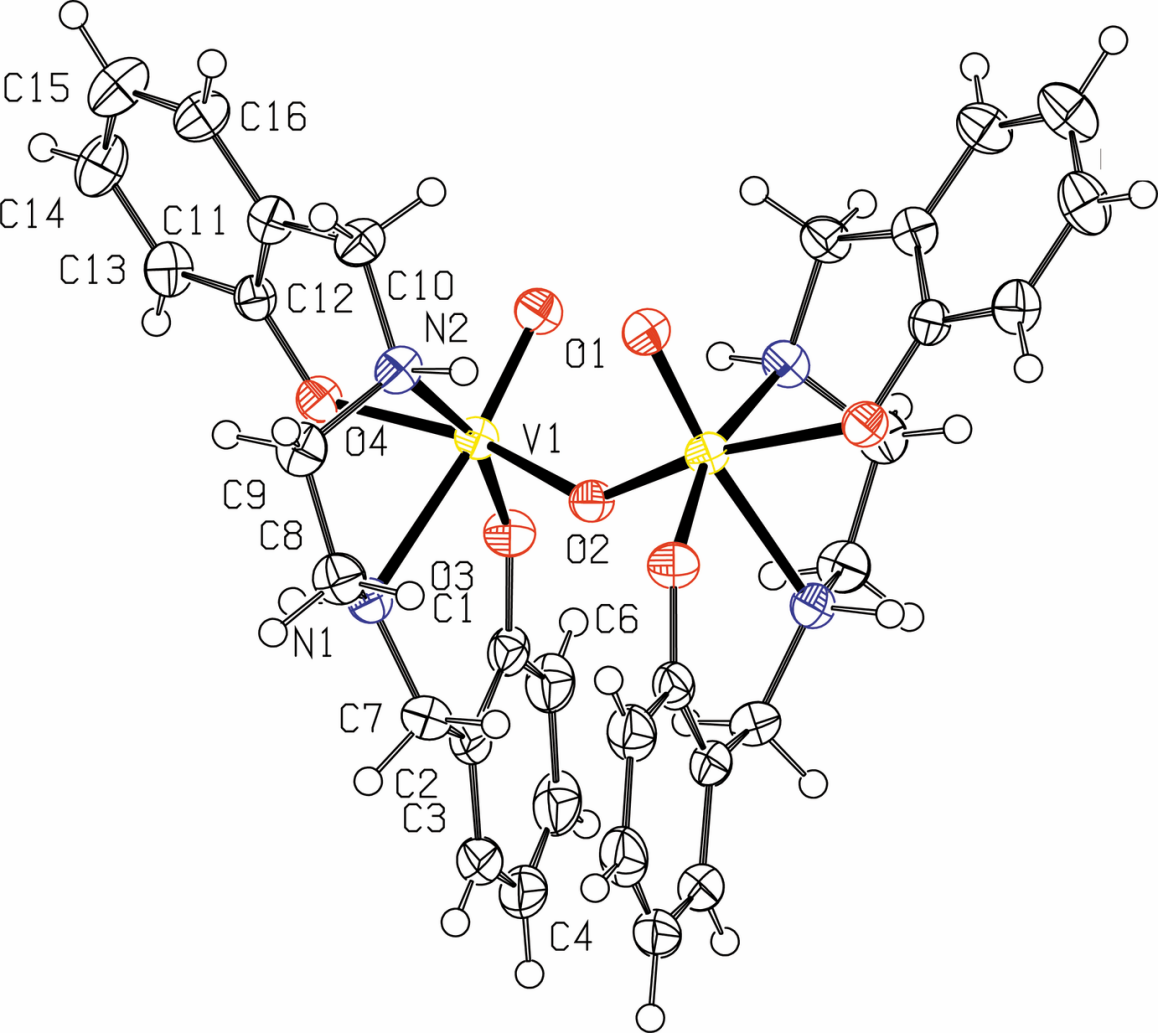
The molecular structure of **1** with 50% probability level ellipsoids. Symmetry code: (_a) 1 − *x*, *y*, 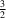 − *z*.

Complex **1** crystallizes in the monoclininc space group *C*2/*c* where the second half of the mol­ecule is symmetry generated (Fig. 1 ▸). The vanadium centers are bridged by one oxygen atom with each vanadium center also having an oxo ligand coordinated. The V=O and V—O_*μ*-oxo_ distances of 1.6134 (14) and 1.8233 (8) Å, respectively, are shorter than the V—O_p_ (p = phenolato) distances of 1.8427 (14) and 1.9235 (14) Å with the V—O_p_ bond *trans* to the *μ*-oxo being longer consistent with a stronger *trans*-effect. The V—O—V bond angle is 140.32 (12)° while the O_oxido_—V—O_*μ*-oxo_ bond angle is 100.03 (7)°. Each vanadium center is coordinated to six atoms, with a highly distorted octa­hedral arrangement. The axial position is defined by the V=O unit with an axial angle of 171.30 (7)° for O1—V1—N1. The axial bond distances of V1—O1 and V1—N1 are 1.6134 (14) and 2.2809 (17) Å, respectively. The equatorial angles deviate from 90° with a O2—V1—O3 angle of 96.56 (6)°, a O3—V1—O4 angle of 92.07 (6)°, a O4—V1—N2 angle of 80.76 (6)°, and an N2—V1—O2 angle of 85.41 (5)°. Combined, the bond distances and angles around the vanadium center reflect a distorted octa­hedral geometry.
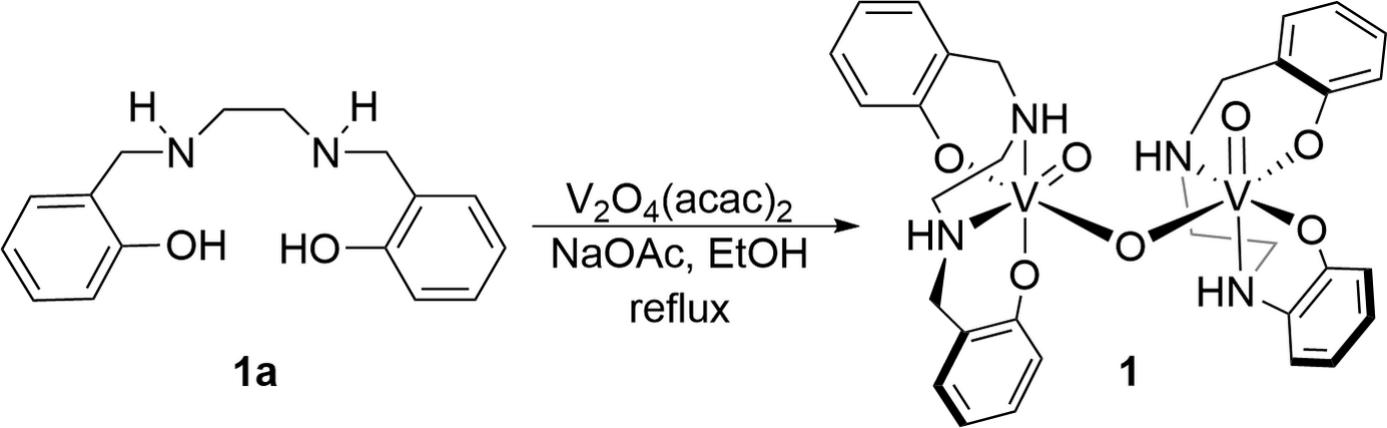


## 3. Supra­molecular features

**Figure 2:**
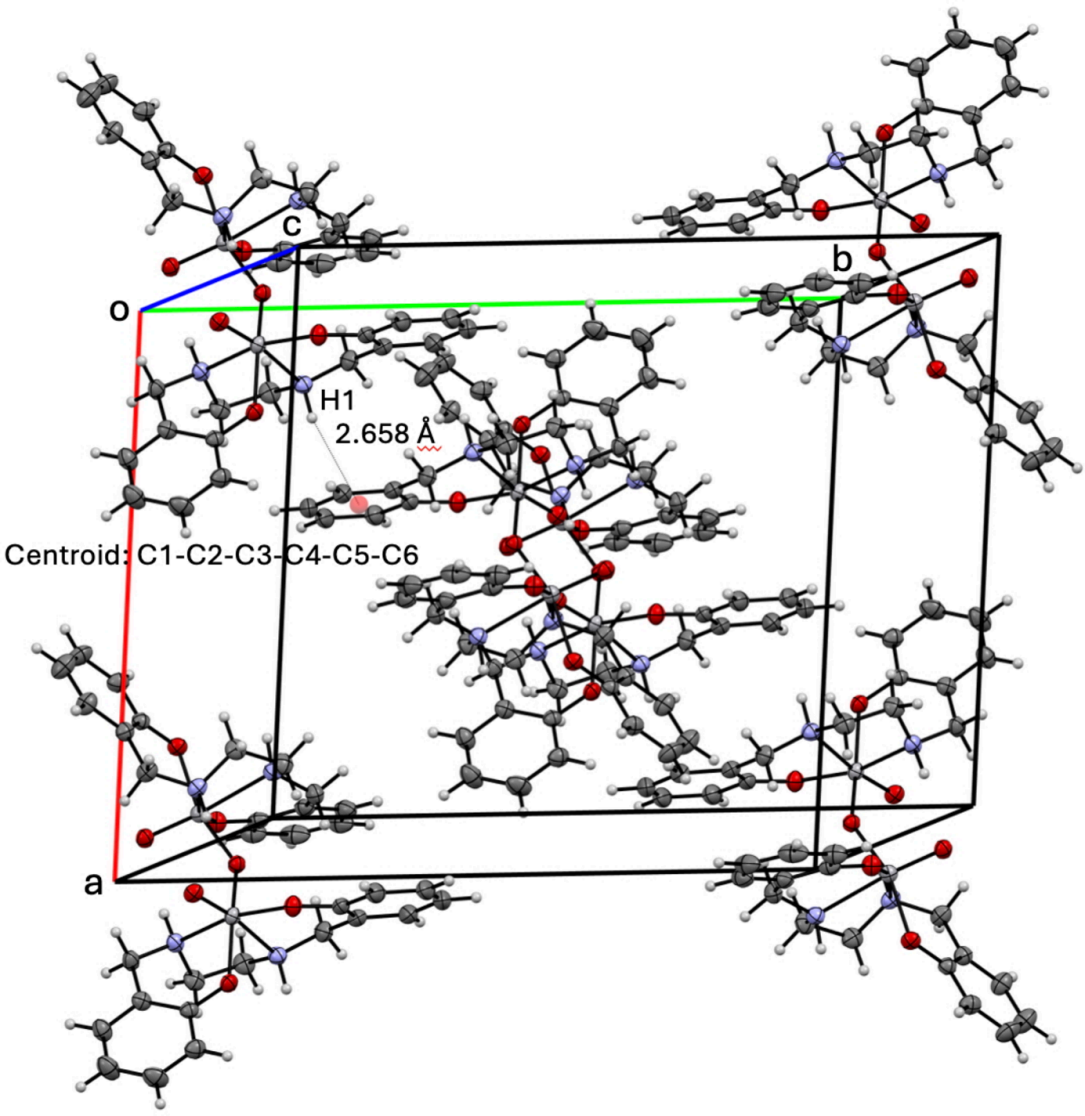
The unit -packing for **1** highlighting inter­molecular π inter­actions.

**Figure 3:**
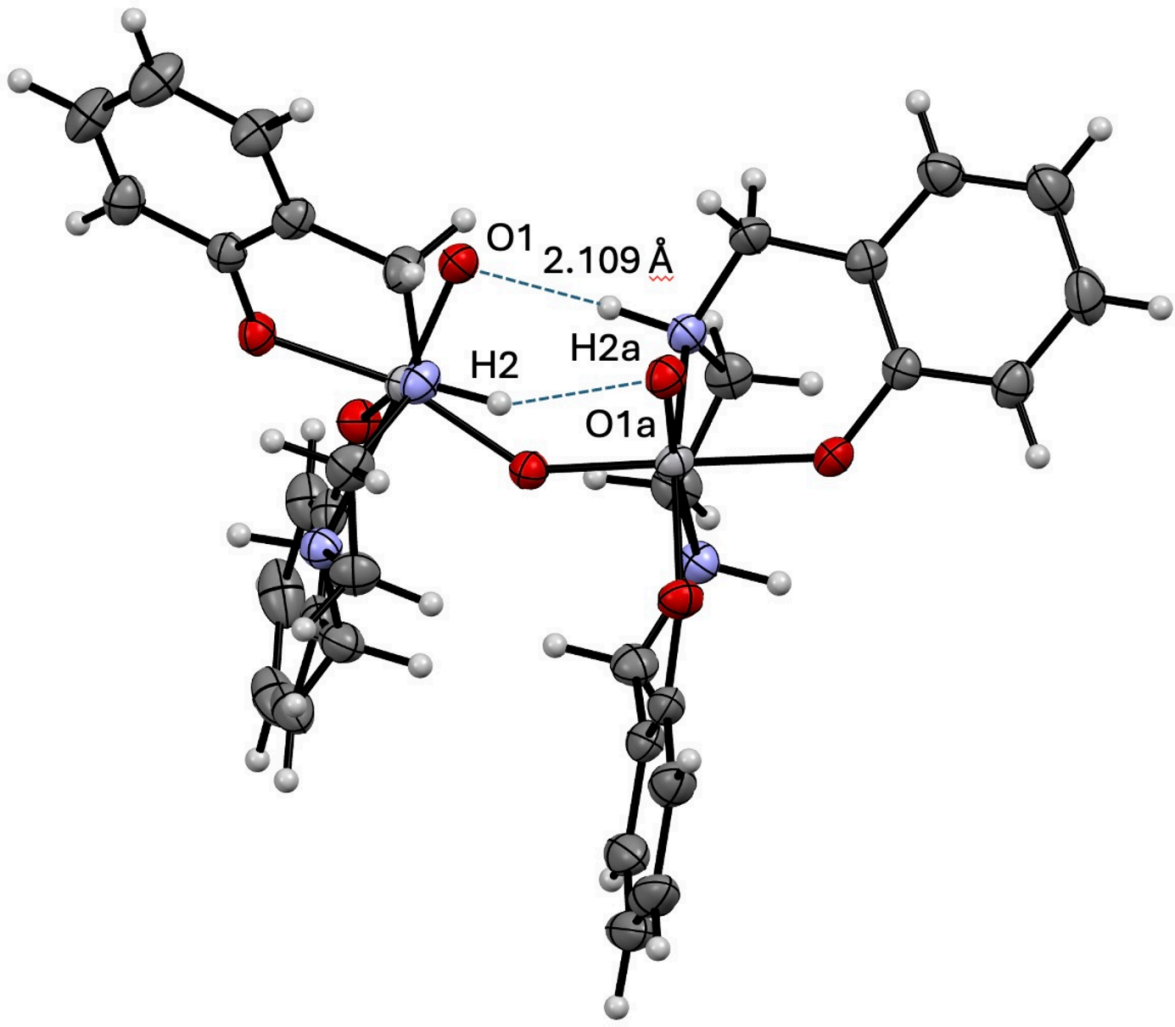
View of μ-oxido-bis­[(2,2′-{[ethane-1,2-diylbis(aza­nedi­yl)]bis­(methyl­ene)}diphenolato)oxidovanadium(V)] (**1**), highlighting intra­molecular hydrogen bonding.

Four mol­ecules of complex **1** are packed within the unit cell, with structural stabilization from inter­molecular π inter­actions with phenyl groups, and intra­molecular hydrogen bonding (Fig. 2 ▸). The π inter­action is found between H1 and the centroid defined by C1–C6 at symmetry position 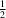 − *y*, 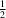 − *y*, 1 − *z* with a distance of 2.66 Å. The closest distance is 2.59 Å between C3⋯H1, which is repeated throughout the packing due to the high level of symmetry in the mol­ecule. Intra­molecular hydrogen bonding is found between H2*a* and O1 with a bond distance of 2.11 Å (Fig. 3 ▸). Distances to hydrogen atoms are reported without standard deviations because the hydrogen atoms were positionally fixed. The H2*a*—N2*a* distance is 1.00 Å, with an O1(1 − *x*, *y*, 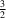 − *z*)⋯N2*a* distance of 2.977 (2) Å and an O1(1 − *x*, *y*, 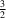 − *z*)⋯H2*a*—N2*a* angle of 144.0°.

## 4. Database survey

A survey of the Cambridge Structural Database (Web accessed August 1, 2025; Groom *et al.*, 2016 ▸) and SciFinder (2025 ▸) yielded no exact matches for complex **1**. However, salan ligands are ubiquitous in coordination chemistry and have been employed to form complexes with metals from across the periodic table. Specifically, oxovanadium complexes of salan ligands have been previously reported including *μ*-oxo dimers (Patra *et al.*, 2021 ▸; Debnath *et al.*, 2018 ▸; Reytman *et al.*, 2012 ▸; Adão *et al.*, 2009 ▸). Complex **1** presents an example of a {OV^v^(*μ*-O)V^v^O} neutral dinuclear *μ*-oxo-bridged vanadium(V) complex featuring a tetra­dentate ligand. The first examples of such complexes based on salan ligands were reported by Correia and coworkers (Adão *et al.*, 2009 ▸). These complexes presented a distorted octa­hedral geometry around the vanadium center and twist-angular configurations with *cis*-orientation between the phenolate O atoms from the salan ligand (*β*-*cis* structure). Complex **1** also displays a *β*-*cis* type arrangement of the phenolate O atoms and the V=O bond distance [1.6134 (14) Å] is comparable to that seen in similar complexes [1.621 (6) Å; Adão *et al.*, 2009 ▸].

## 5. Synthesis and crystallization

The salan ligand precursor (**1a**) used in this study was synthesized by the reductive amination reaction between salicyl­aldehyde and 1,2-ethyl­enedi­amine in a 79% yield. (Wagner *et al.*, 2024 ▸) Complexation was achieved by the reaction of **1a** with the vanadium precursor [V_2_O_4_(acac)_2_] in ethanol at reflux under a N_2_ atmosphere. Complex **1** formed as a dark precipitate and was collected by gravity filtration (77% yield). The resulting dark-purple colored filtrate was stored at room temperature to obtain crystals of the *μ*-oxo vanadium(V) dimer complex (**1**) by slow evaporation. ESI-MS analysis of the crystals exhibited the monomer unit [LV^v^(O)]^+^ at *m*/*z* = 337. Elemental analysis of the precipitate matched a Na[dioxo(*L*)vanadate] complex. We hypothesize that the vanadate complex is in equilibrium with the *μ*-oxo dimer which crystallized out of an ethano­lic solution.

Synthesis of **1** [LV^v^(O)–(*μ*-O)–LV^v^(O)]: In a round-bottom flask, **1a** (0.622 g, 2.28 mmol), sodium acetate (0.761 g, 9.28 mmol), and V_2_O_4_(acac)_2_ (0.417 g, 1.14 mmol) were dissolved in 10 mL of ethanol under a nitro­gen atmosphere. The mixture was heated to reflux for 2 h. After heating, the mixture was cooled down to room temperature. A dark precipitate formed and it was isolated by gravity filtration. The precipitate was washed with cold methanol and dried overnight under a high vacuum to obtain **1** as a dark solid (0.760 g, 77%). The resulting filtrate was stored at room temperature for slow evaporation and produced dark green crystals of **1** in a few days. FTIR: 3266 (N—H), 942 cm^−1^ (V=O). ESI-MS (+ve): *m*/*z* = 337 [C_16_H_18_N_2_O_3_V]^+^. Elemental analysis for calculated C_16_H_18_N_2_O_4_VNa·3 H_2_O: C: 44.66; H: 5.62; N: 6.51. Found: C: 44.61; H: 4.70; N: 5.40.

## 6. Refinement

Crystal data, data collection and structure refinement details are summarized in Table 1 ▸. Hydrogen atoms were placed in calculated positions using the AFIX command in *SHELXL* and refined using a riding model.

## Supplementary Material

Crystal structure: contains datablock(s) I. DOI: 10.1107/S2056989025008631/tx2104sup1.cif

Structure factors: contains datablock(s) I. DOI: 10.1107/S2056989025008631/tx2104Isup3.hkl

CCDC reference: 2492548

Additional supporting information:  crystallographic information; 3D view; checkCIF report

## Figures and Tables

**Table 1:** Experimental details

Crystal data	
Chemical formula	[V_2_(C_16_H_18_N_2_O_2_)_2_O_3_]
*M*_r_	690.53
Crystal system, space group	Monoclinic, *C*2/*c*
Temperature (K)	150
*a*, *b*, *c* (Å)	15.5603 (11), 20.7953 (11), 10.4197 (6)
β (°)	107.622 (3)
*V* (Å^3^)	3213.4 (3)
*Z*	4
Radiation type	Mo *K*α
μ (mm^−1^)	0.63
Crystal size (mm)	0.2 × 0.15 × 0.05

Data collection	
Diffractometer	Bruker Venture Kappa D8
Absorption correction	Multi-scan (*SADABS*; Krause *et al.*, 2015 ▸)
*T*_min_, *T*_max_	0.609, 0.746
No. of measured, independent and observed [*I* > 2σ(*I*)] reflections	7304, 3713, 3253
*R*_int_	0.038
(sin θ/λ)_max_ (Å^−1^)	0.651

Refinement	
*R*[*F*^2^ > 2σ(*F*^2^)], *wR*(*F*^2^), *S*	0.043, 0.100, 1.08
No. of reflections	3713
No. of parameters	204
H-atom treatment	H-atom parameters constrained
Δρ_max_, Δρ_min_ (e Å^−3^)	0.50, −0.42

Computer programs: *APEX5* (Bruker, 2023 ▸), *SAINT* (Bruker, 2020 ▸), *SHELXT* (Sheldrick, 2015*a* ▸), *SHELXL2019/2* (Sheldrick, 2015*b* ▸) and *OLEX2* (Dolomanov *et al.*, 2009 ▸).

## supplementary crystallographic information

### µ-Oxido-bis[(2,2'-{[ethane-1,2-diylbis(azanediyl)]bis(methylene)}diphenolato)oxidovanadium(V)] Crystal data

**Table:**

[V_2_(C_16_H_18_N_2_O_2_)_2_O_3_]	*F*(000) = 1432
*M**_r_* = 690.53	*D*_x_ = 1.427 Mg m^−^^3^
Monoclinic, *C*2/*c*	Mo *K*α radiation, λ = 0.71073 Å
*a* = 15.5603 (11) Å	Cell parameters from 9335 reflections
*b* = 20.7953 (11) Å	θ = 2.3–27.3°
*c* = 10.4197 (6) Å	µ = 0.63 mm^−^^1^
β = 107.622 (3)°	*T* = 150 K
*V* = 3213.4 (3) Å^3^	Prism, green
*Z* = 4	0.2 × 0.15 × 0.05 mm

### µ-Oxido-bis[(2,2'-{[ethane-1,2-diylbis(azanediyl)]bis(methylene)}diphenolato)oxidovanadium(V)] Data collection

**Table:**

Bruker Venture Kappa D8 diffractometer	3253 reflections with *I* > 2σ(*I*)
φ and ω scans	*R*_int_ = 0.038
Absorption correction: multi-scan (SADABS; Krause *et al.*, 2015)	θ_max_ = 27.6°, θ_min_ = 2.3°
*T*_min_ = 0.609, *T*_max_ = 0.746	*h* = −20→15
7304 measured reflections	*k* = −16→27
3713 independent reflections	*l* = −13→13

### µ-Oxido-bis[(2,2'-{[ethane-1,2-diylbis(azanediyl)]bis(methylene)}diphenolato)oxidovanadium(V)] Refinement

**Table:**

Refinement on *F*^2^	Primary atom site location: structure-invariant direct methods
Least-squares matrix: full	Hydrogen site location: inferred from neighbouring sites
*R*[*F*^2^ > 2σ(*F*^2^)] = 0.043	H-atom parameters constrained
*wR*(*F*^2^) = 0.100	*w* = 1/[σ^2^(*F*_o_^2^) + (0.0367*P*)^2^ + 5.1463*P*] where *P* = (*F*_o_^2^ + 2*F*_c_^2^)/3
*S* = 1.08	(Δ/σ)_max_ = 0.001
3713 reflections	Δρ_max_ = 0.50 e Å^−^^3^
204 parameters	Δρ_min_ = −0.41 e Å^−^^3^
0 restraints	

### µ-Oxido-bis[(2,2'-{[ethane-1,2-diylbis(azanediyl)]bis(methylene)}diphenolato)oxidovanadium(V)] Special details

**Table:**

Geometry. All esds (except the esd in the dihedral angle between two l.s. planes) are estimated using the full covariance matrix. The cell esds are taken into account individually in the estimation of esds in distances, angles and torsion angles; correlations between esds in cell parameters are only used when they are defined by crystal symmetry. An approximate (isotropic) treatment of cell esds is used for estimating esds involving l.s. planes.

### µ-Oxido-bis[(2,2'-{[ethane-1,2-diylbis(azanediyl)]bis(methylene)}diphenolato)oxidovanadium(V)] Fractional atomic coordinates and isotropic or equivalent isotropic displacement parameters (Å^2^)

**Table:**

	*x*	*y*	*z*	*U*_iso_*/*U*_eq_	
V1	0.39689 (2)	0.41197 (2)	0.62885 (3)	0.02216 (11)	
O1	0.43353 (9)	0.47851 (7)	0.58524 (14)	0.0269 (3)	
O2	0.500000	0.38221 (9)	0.750000	0.0241 (4)	
O3	0.39756 (10)	0.35637 (7)	0.49145 (14)	0.0284 (3)	
O4	0.27065 (9)	0.43015 (7)	0.54986 (14)	0.0258 (3)	
N1	0.33431 (12)	0.32747 (8)	0.70801 (17)	0.0266 (4)	
H1	0.269592	0.324980	0.652277	0.032*	
N2	0.36955 (12)	0.45416 (8)	0.79859 (17)	0.0261 (4)	
H2	0.428501	0.455870	0.871711	0.031*	
C1	0.39372 (13)	0.29241 (11)	0.4713 (2)	0.0282 (5)	
C2	0.38266 (14)	0.24750 (10)	0.5653 (2)	0.0291 (5)	
C3	0.38420 (15)	0.18215 (11)	0.5338 (3)	0.0380 (6)	
H3	0.377546	0.151094	0.597010	0.046*	
C4	0.39509 (16)	0.16141 (12)	0.4138 (3)	0.0440 (6)	
H4	0.396727	0.116724	0.395741	0.053*	
C5	0.40354 (16)	0.20579 (13)	0.3209 (3)	0.0446 (6)	
H5	0.409930	0.191767	0.237450	0.054*	
C6	0.40278 (15)	0.27119 (12)	0.3483 (2)	0.0371 (5)	
H6	0.408434	0.301655	0.283367	0.045*	
C7	0.37424 (15)	0.26437 (10)	0.7020 (2)	0.0317 (5)	
H7A	0.336911	0.231242	0.727944	0.038*	
H7B	0.434944	0.263112	0.768910	0.038*	
C8	0.33584 (16)	0.34377 (11)	0.8470 (2)	0.0330 (5)	
H8A	0.397075	0.336843	0.909913	0.040*	
H8B	0.293264	0.315829	0.875163	0.040*	
C9	0.30904 (15)	0.41335 (11)	0.8504 (2)	0.0312 (5)	
H9A	0.245738	0.419340	0.794063	0.037*	
H9B	0.313738	0.426014	0.943839	0.037*	
C10	0.33729 (14)	0.52175 (10)	0.7745 (2)	0.0288 (5)	
H10A	0.385972	0.548440	0.759261	0.035*	
H10B	0.325105	0.538053	0.856602	0.035*	
C11	0.25374 (14)	0.52991 (10)	0.6570 (2)	0.0266 (4)	
C12	0.22502 (13)	0.48466 (10)	0.5526 (2)	0.0242 (4)	
C13	0.14539 (14)	0.49649 (12)	0.4483 (2)	0.0313 (5)	
H13	0.124709	0.465859	0.378091	0.038*	
C14	0.09655 (16)	0.55215 (13)	0.4464 (2)	0.0409 (6)	
H14	0.042555	0.559364	0.375081	0.049*	
C15	0.12568 (17)	0.59750 (13)	0.5475 (2)	0.0430 (6)	
H15	0.092928	0.636278	0.544961	0.052*	
C16	0.20313 (16)	0.58549 (11)	0.6523 (2)	0.0340 (5)	
H16	0.222328	0.616015	0.723077	0.041*	

### µ-Oxido-bis[(2,2'-{[ethane-1,2-diylbis(azanediyl)]bis(methylene)}diphenolato)oxidovanadium(V)] Atomic displacement parameters (Å^2^)

**Table:**

	*U*^11^	*U*^22^	*U*^33^	*U*^12^	*U*^13^	*U*^23^
V1	0.02398 (18)	0.02299 (19)	0.01842 (17)	−0.00135 (13)	0.00478 (13)	−0.00123 (13)
O1	0.0274 (7)	0.0273 (8)	0.0236 (7)	−0.0011 (6)	0.0042 (6)	0.0024 (6)
O2	0.0246 (10)	0.0222 (10)	0.0240 (10)	0.000	0.0050 (8)	0.000
O3	0.0335 (8)	0.0279 (8)	0.0229 (7)	−0.0009 (6)	0.0073 (6)	−0.0031 (6)
O4	0.0248 (7)	0.0269 (7)	0.0231 (7)	−0.0015 (6)	0.0035 (6)	−0.0035 (6)
N1	0.0266 (9)	0.0261 (9)	0.0282 (9)	−0.0001 (7)	0.0099 (7)	0.0008 (7)
N2	0.0268 (9)	0.0287 (9)	0.0208 (8)	0.0027 (7)	0.0042 (7)	−0.0019 (7)
C1	0.0206 (10)	0.0308 (11)	0.0307 (11)	−0.0003 (8)	0.0043 (8)	−0.0086 (9)
C2	0.0217 (10)	0.0281 (11)	0.0364 (12)	−0.0029 (8)	0.0068 (9)	−0.0049 (9)
C3	0.0279 (11)	0.0293 (12)	0.0572 (15)	−0.0028 (9)	0.0135 (11)	−0.0076 (11)
C4	0.0303 (12)	0.0329 (13)	0.0693 (18)	−0.0028 (10)	0.0159 (12)	−0.0198 (13)
C5	0.0315 (12)	0.0508 (16)	0.0537 (15)	−0.0040 (11)	0.0160 (11)	−0.0275 (13)
C6	0.0313 (12)	0.0435 (14)	0.0372 (12)	−0.0016 (10)	0.0114 (10)	−0.0121 (11)
C7	0.0336 (11)	0.0250 (11)	0.0369 (12)	−0.0025 (9)	0.0115 (10)	0.0013 (9)
C8	0.0389 (12)	0.0345 (12)	0.0285 (11)	0.0004 (10)	0.0147 (10)	0.0037 (9)
C9	0.0349 (12)	0.0376 (13)	0.0233 (10)	0.0017 (10)	0.0121 (9)	0.0000 (9)
C10	0.0300 (11)	0.0288 (11)	0.0243 (10)	0.0026 (9)	0.0034 (8)	−0.0061 (9)
C11	0.0277 (10)	0.0304 (11)	0.0224 (10)	0.0014 (9)	0.0083 (8)	0.0015 (8)
C12	0.0233 (9)	0.0267 (11)	0.0233 (10)	−0.0009 (8)	0.0079 (8)	0.0006 (8)
C13	0.0286 (11)	0.0388 (13)	0.0246 (10)	−0.0010 (9)	0.0053 (9)	−0.0015 (9)
C14	0.0318 (12)	0.0515 (16)	0.0346 (12)	0.0129 (11)	0.0028 (10)	0.0051 (11)
C15	0.0444 (14)	0.0453 (15)	0.0364 (13)	0.0172 (12)	0.0079 (11)	0.0006 (11)
C16	0.0394 (12)	0.0336 (12)	0.0285 (11)	0.0069 (10)	0.0093 (9)	−0.0022 (9)

### µ-Oxido-bis[(2,2'-{[ethane-1,2-diylbis(azanediyl)]bis(methylene)}diphenolato)oxidovanadium(V)] Geometric parameters (Å, º)

**Table:**

V1—O1	1.6134 (14)	C5—C6	1.390 (3)
V1—O2	1.8233 (8)	C6—H6	0.9500
V1—O3	1.8427 (14)	C7—H7A	0.9900
V1—O4	1.9235 (14)	C7—H7B	0.9900
V1—N1	2.2809 (17)	C8—H8A	0.9900
V1—N2	2.1295 (17)	C8—H8B	0.9900
O3—C1	1.345 (3)	C8—C9	1.509 (3)
O4—C12	1.343 (2)	C9—H9A	0.9900
N1—H1	1.0000	C9—H9B	0.9900
N1—C7	1.461 (3)	C10—H10A	0.9900
N1—C8	1.481 (3)	C10—H10B	0.9900
N2—H2	1.0000	C10—C11	1.502 (3)
N2—C9	1.485 (3)	C11—C12	1.405 (3)
N2—C10	1.488 (3)	C11—C16	1.391 (3)
C1—C2	1.401 (3)	C12—C13	1.401 (3)
C1—C6	1.403 (3)	C13—H13	0.9500
C2—C3	1.400 (3)	C13—C14	1.381 (3)
C2—C7	1.511 (3)	C14—H14	0.9500
C3—H3	0.9500	C14—C15	1.384 (4)
C3—C4	1.381 (4)	C15—H15	0.9500
C4—H4	0.9500	C15—C16	1.382 (3)
C4—C5	1.373 (4)	C16—H16	0.9500
C5—H5	0.9500		
			
O1—V1—O2	100.03 (7)	C1—C6—H6	119.8
O1—V1—O3	103.52 (7)	C5—C6—C1	120.3 (2)
O1—V1—O4	96.49 (7)	C5—C6—H6	119.8
O1—V1—N1	171.30 (7)	N1—C7—C2	114.10 (18)
O1—V1—N2	93.27 (7)	N1—C7—H7A	108.7
O2—V1—O3	96.56 (6)	N1—C7—H7B	108.7
O2—V1—O4	158.99 (5)	C2—C7—H7A	108.7
O2—V1—N1	82.57 (6)	C2—C7—H7B	108.7
O2—V1—N2	85.41 (5)	H7A—C7—H7B	107.6
O3—V1—O4	92.07 (6)	N1—C8—H8A	110.0
O3—V1—N1	84.32 (6)	N1—C8—H8B	110.0
O3—V1—N2	162.44 (7)	N1—C8—C9	108.62 (18)
O4—V1—N1	79.26 (6)	H8A—C8—H8B	108.3
O4—V1—N2	80.76 (6)	C9—C8—H8A	110.0
N2—V1—N1	78.61 (7)	C9—C8—H8B	110.0
V1—O2—V1^i^	140.32 (12)	N2—C9—C8	109.24 (17)
C1—O3—V1	136.97 (14)	N2—C9—H9A	109.8
C12—O4—V1	129.42 (12)	N2—C9—H9B	109.8
V1—N1—H1	107.2	C8—C9—H9A	109.8
C7—N1—V1	116.28 (13)	C8—C9—H9B	109.8
C7—N1—H1	107.2	H9A—C9—H9B	108.3
C7—N1—C8	111.48 (17)	N2—C10—H10A	108.7
C8—N1—V1	107.17 (13)	N2—C10—H10B	108.7
C8—N1—H1	107.2	N2—C10—C11	114.12 (17)
V1—N2—H2	106.2	H10A—C10—H10B	107.6
C9—N2—V1	111.98 (13)	C11—C10—H10A	108.7
C9—N2—H2	106.2	C11—C10—H10B	108.7
C9—N2—C10	112.48 (16)	C12—C11—C10	123.35 (18)
C10—N2—V1	113.04 (12)	C16—C11—C10	117.61 (19)
C10—N2—H2	106.2	C16—C11—C12	119.05 (19)
O3—C1—C2	124.03 (19)	O4—C12—C11	122.41 (18)
O3—C1—C6	116.2 (2)	O4—C12—C13	118.75 (18)
C2—C1—C6	119.8 (2)	C13—C12—C11	118.84 (19)
C1—C2—C7	124.62 (19)	C12—C13—H13	119.6
C3—C2—C1	118.0 (2)	C14—C13—C12	120.8 (2)
C3—C2—C7	117.3 (2)	C14—C13—H13	119.6
C2—C3—H3	119.0	C13—C14—H14	119.7
C4—C3—C2	122.1 (2)	C13—C14—C15	120.5 (2)
C4—C3—H3	119.0	C15—C14—H14	119.7
C3—C4—H4	120.2	C14—C15—H15	120.5
C5—C4—C3	119.6 (2)	C16—C15—C14	119.0 (2)
C5—C4—H4	120.2	C16—C15—H15	120.5
C4—C5—H5	119.9	C11—C16—H16	119.1
C4—C5—C6	120.3 (2)	C15—C16—C11	121.8 (2)
C6—C5—H5	119.9	C15—C16—H16	119.1
			
V1—O3—C1—C2	3.4 (3)	C1—C2—C3—C4	−0.8 (3)
V1—O3—C1—C6	−175.72 (15)	C1—C2—C7—N1	29.0 (3)
V1—O4—C12—C11	−26.4 (3)	C2—C1—C6—C5	−1.9 (3)
V1—O4—C12—C13	154.26 (15)	C2—C3—C4—C5	−0.9 (4)
V1—N1—C7—C2	−51.4 (2)	C3—C2—C7—N1	−154.50 (19)
V1—N1—C8—C9	41.95 (19)	C3—C4—C5—C6	1.2 (4)
V1—N2—C9—C8	40.7 (2)	C4—C5—C6—C1	0.2 (4)
V1—N2—C10—C11	57.6 (2)	C6—C1—C2—C3	2.2 (3)
O1—V1—O2—V1^i^	30.44 (5)	C6—C1—C2—C7	178.6 (2)
O1—V1—O3—C1	163.55 (19)	C7—N1—C8—C9	170.24 (17)
O2—V1—O3—C1	61.6 (2)	C7—C2—C3—C4	−177.5 (2)
O3—V1—O2—V1^i^	135.47 (5)	C8—N1—C7—C2	−174.60 (17)
O3—C1—C2—C3	−176.9 (2)	C9—N2—C10—C11	−70.4 (2)
O3—C1—C2—C7	−0.5 (3)	C10—N2—C9—C8	169.30 (17)
O3—C1—C6—C5	177.2 (2)	C10—C11—C12—O4	−0.2 (3)
O4—V1—O2—V1^i^	−110.90 (19)	C10—C11—C12—C13	179.2 (2)
O4—V1—O3—C1	−99.3 (2)	C10—C11—C16—C15	179.4 (2)
O4—C12—C13—C14	−179.5 (2)	C11—C12—C13—C14	1.1 (3)
N1—V1—O2—V1^i^	−141.16 (4)	C12—C11—C16—C15	−0.3 (3)
N1—V1—O3—C1	−20.27 (19)	C12—C13—C14—C15	0.2 (4)
N1—C8—C9—N2	−55.6 (2)	C13—C14—C15—C16	−1.6 (4)
N2—V1—O2—V1^i^	−62.07 (5)	C14—C15—C16—C11	1.7 (4)
N2—V1—O3—C1	−34.0 (3)	C16—C11—C12—O4	179.58 (19)
N2—C10—C11—C12	−20.3 (3)	C16—C11—C12—C13	−1.1 (3)
N2—C10—C11—C16	159.96 (19)		

Symmetry code: (i) −*x*+1, *y*, −*z*+3/2.
